# Age Differences in Neural Response to Stereotype Threat and Resiliency for Self-Referenced Information

**DOI:** 10.3389/fnhum.2013.00537

**Published:** 2013-09-06

**Authors:** Gabriel Colton, Eric D. Leshikar, Angela H. Gutchess

**Affiliations:** ^1^Department of Psychology, Brandeis University, Waltham, MA, USA

**Keywords:** aging, stereotypes, fMRI, self-referencing, cortical midline regions, stereotype threat, cognition

## Abstract

To investigate the contribution of cortical midline regions to stereotype threat and resiliency, we compared age groups in an event-related functional MRI study. During scanning, 17 younger and 16 older adults judged whether words stereotypical of aging and control words described them. Judging stereotype words versus control words revealed higher activations in posterior midline regions associated with self-referencing, including the precuneus, for older adults compared to younger adults. While heightening salience of stereotypes can evoke a threat response, detrimentally affecting performance, invoking stereotypes can also lead to a phenomenon called resilience, where older adults use those stereotypes to create downward social-comparisons to “other” older adults and elevate their own self-perception. In an exploration of brain regions underlying stereotype threat responses as well as resilience responses, we found significant activation in older adults for threat over resilient responses in posterior midline regions including the precuneus, associated with self-reflective thought, and parahippocampal gyrus, implicated in autobiographical memory. These findings have implications for understanding how aging stereotypes may affect the engagement of regions associated with contextual and social processing of self-relevant information, indicating ways in which stereotype threat can affect the engagement of neural resources with age.

## Introduction

Stereotypes represent shared beliefs that save time and energy by allowing one to judge other people based on group membership rather than on the basis of their complex and unique personalities (McGarty et al., 2002). The development and maintenance of aging-related stereotypes is unique relative to other group-related stereotypes for several reasons. First, older adults are the only stigmatized group that transitions from an out-group (i.e., as they are seen by young adults) to an inevitable in-group as those young adults reach old age. Second, there is initially no reason for younger adults to defend against negative aging-related stereotypes as they only apply to others. With aging, individuals may begin to internalize and become susceptible to aging-related stereotypes (Levy and Banaji, 2002). Third, negative aging-related stereotypes are perpetuated and are present across cultures (Cuddy et al., 2005) and held by both younger and older adults alike (Boduroglu et al., 2006), illustrating the potential far-reaching implications of aging-related stereotype research.

Aging-related stereotypes can be both positive (e.g., wise, accomplished, enlightened, and respected) and negative (e.g., forgetful, slow, confused, and inept) (Mueller et al., 1986; Levy, 2003). While stereotypes are useful to the extent that they can help direct interactions with others from different groups, they can also be detrimental under some circumstances. Steele and Aronson’s (1995) seminal work on stereotype threat exemplified this by demonstrating that African American students’ performance on a personally important ability (academic performance) was impaired when they were primed to think about relevant negative stereotypical information prior to the task. African American students performed similarly to their white counterparts under non-threatening conditions, suggesting that the performance decrement seen under threatening conditions was not indicative of the students’ actual abilities. Studies in older adults have also demonstrated the negative impact of stereotype threat on cognitive function (e.g., memory), psychomotor function (e.g., walking rate and handwriting), physiological factors (e.g., heart rate and blood pressure), and self-worth (e.g., will to live) compared to older adults who do not experience a threat manipulation (Bargh et al., 1996; Horton et al., 2008, 2010; for a review see Levy, 2003).

In addition to how much value one places on the ability being measured (Hess et al., 2003), such that stereotype threat conditions are more detrimental to individuals who place high importance on the stereotyped ability, the self-relevance of a stereotype influences the extent to which stereotype threat impacts performance (Shih et al., 2002). To date, no extant work has investigated how the self-relevance of stereotypes impacts neural response^1^. The social cognitive network (for a review, see Lieberman, 2007; Van Overwalle, 2009), encompassing regions including medial prefrontal cortex and temporo-parietal junction, is broadly implicated in stereotype-relevant processes. The network is thought to underlie evaluative processing, mentalizing about others, and making social rather than non-social judgments (Quadflieg et al., 2009, 2011; Quadflieg and Macrae, 2011). This same network is also implicated in thinking about the self and uniquely supports memory enhancements for self-relevant information (Rogers et al., 1977; Symons and Johnson, 1997; Kelley et al., 2002; Fossati et al., 2004; Macrae et al., 2004). Given the overlapping networks involved in self-referencing and stereotyping, as well as the self-relevance of aging-related stereotypes over the lifespan, the intersection of these topics offers a way to explore the self-relevant processes evoked by stereotypes across age groups.

The cortical midline network implicated in self-referencing, as well as stereotyping, has been divided into distinct subcomponents on the basis of meta-analysis and functional task dissociations. Northoff et al. (2006) propose that ventral anterior regions [including ventral medial prefrontal and anterior cingulate cortex (ACC)] are responsible for coding self-referentiality of information, dorsal anterior regions (including dorsal medial prefrontal cortex) may reflect the evaluative components of self-referencing, particularly compared to other stimuli or persons, and that posterior midline regions [including precuneus and posterior cingulate (PCC)] potentially reflect “self in context,” including autobiographical memory. Another distinction separates anterior regions (medial prefrontal cortex; mPFC), engaged during more inward-focused thought, from posterior regions (PCC; lingual gyrus), reflecting a more outward-directed, social and contextual focus, on the basis of response to different types of goals (Johnson et al., 2006; Mitchell et al., 2009). MPFC and PCC can also be distinguished on the basis of thinking about internal (i.e., character traits) and external (i.e., appearance) features of self and other (Moran et al., 2011).

The present study investigates the effects of aging on the recruitment of cortical midline regions during self-relevance judgments about words related to age-related stereotypes, compared to control words. To test this, we created a set of positive and negative trait adjectives, some of which are stereotypical of older adults (e.g., wise, frail) and some that are not stereotypical of either age group (e.g., friendly, irrational). Both younger and older participants judged the self-descriptiveness of these words.

Due to the greater self-relevance of age-related stereotypes, we expect greater activity in anterior and posterior midline regions in older than in younger adults for stereotyped relative to control words. Both younger and older adults have been shown to engage mPFC and mid-cingulate during judgments of self-relevance (Gutchess et al., 2007; see age differences during successful encoding in Gutchess et al., 2010) and mPFC when making judgments about same- versus other-age individuals (Ebner et al., 2011). Moreover, mPFC and PCC activity in younger adults increases linearly with increasing self-relevance of stimuli (Moran et al., 2006, 2009), suggesting that highly self-relevant words engage this region more than less or non-self-relevant words. Because stereotyped words may apply more to older versus younger adults, we predicted that older adults would engage regions implicated in self-relevance (e.g., mPFC, PCC) more than young in response to stereotyped words versus control words. We additionally anticipated that this relationship would be magnified for stereotyped words *endorsed* by participants as self-relevant, compared to non-endorsed words.

While cortical midlines regions are considered to be part of the “default network” broadly implicated in social cognition but deactivated during tasks demanding external attention, it is important to consider the effects of aging on this network during tasks thought to rely on this network (rather than on the *suppression* of this network). Some studies of aging report that this network is disrupted with aging, including during self versus non-self-judgments (Grady et al., 2012). These changes could reflect different strategies, types of processes, or foci across age groups during tasks (Grady et al., 2012), and thus it is important to consider the response of the network to various types of content. The engagement of cortical midline regions is affected by aging as a function of thinking about different self-relevant agendas, such as hopes (e.g., aspirations for career success) and duties (e.g., obligation to care for parents or grandchildren) (Johnson et al., 2006; Mitchell et al., 2009). While activity in both anterior (e.g., mPFC) and posterior (e.g., PCC) cortical midline regions is attenuated with age, the age difference is exaggerated for anterior regions. Engagement of anterior regions reflects thinking about hopes and aspirations, and this is considered to be less of a motivational focus for older adults (Mitchell et al., 2009). Instead, older adults may place more focus on duties and obligations, consistent with post-task reports and more intact engagement of posterior regions (Mitchell et al., 2009). These differences potentially indicate more age-related changes to an inward self-focus, reflected by anterior regions, than to an outward self-focus, governed by posterior regions (Johnson et al., 2006; Mitchell et al., 2009; see also Northoff et al., 2006).

This distinction allows for the possibility that the ways in which older adults make self-relevant judgments may be more contextual and social, particularly for stereotyped information, compared to younger adults. This may occur because age-related stereotypes may reflect limitations in how one achieves goals and fulfills obligations. The overlap between self-referencing and age-related stereotypes is intriguing due to potential differences in the extent to which an individual sees him or herself as a member of the target group, conforming to the stereotypes. Older adults have more complex and varied views of the typical older adult than do younger adults (Hummert, 2011). This is consistent with the out-group homogeneity effect, which posits that people view out-group members as more similar to each other than in-group members (Park and Rothbart, 1982). Interestingly, while presenting older adults with negative age-related stereotypical information may lead to an increasingly negative peer-perception, it also can lead to an increasingly positive self-perception (Pinquart, 2002). When reflecting on age-related stereotypes, older adults created an additional out-group of “other old people.” By projecting the negative stereotypes onto that group rather than themselves, older adults reduced the personal relevancy of the stereotype. This phenomenon, termed “resiliency” (Pinquart, 2002), stood in contrast to previous studies showing that negative stereotypes (threatening conditions) adversely affected self-concept as well as performance (for a review see Levy, 2003; see Meisner, 2012 for a meta-analysis). The current study will investigate resiliency within our self-relevance paradigm by separating words reflecting a positive self-image (endorsed words reflecting positive aging stereotypes and denied, or non-endorsed, words reflecting negative aging stereotypes) from words signifying a threat response (endorsed words reflecting negative aging stereotypes and denied words reflecting positive aging stereotypes). Given that participants will view these words without being explicitly aware of the presence of negative age-related stereotypes, we may have more sensitivity to detect age differences, as overtly directing older adults toward specific stereotypes may allow them to try to actively resist them (Hess et al., 2004).

Combined with our expectation that older adults will have a more heterogeneous perspective on same age peers than young, we hypothesize that older adults’ judgments of self-relevance of traits will have a more social-comparison focus (e.g., downward social-comparison, such that one compares favorably to peers) than younger adults, particularly for aging-related stereotypes (Johnson et al., 2006; Mitchell et al., 2009). Specifically, this should be reflected in older adults’ increased activation of posterior midline regions (PCC/precuneus), compared to younger adults, when self-relevance judgments are made for stereotyped versus control words. If older adults process age-related stereotype information in a manner oriented to social context, this outward focus could lead to a threatened response (e.g., via salience of negative aging stereotypes) or a resilient response (e.g., via downward social-comparisons).

Previous research on stereotype threat reveals that emotional and control processes are invoked under threat. In response to gender-based stereotype threat conditions, activation increased in ventral anterior cingulate cortex (vACC) (Krendl et al., 2008) and the amygdala (Wraga et al., 2007), regions implicated in the evaluation and regulation of emotion (Bush et al., 2000). Given the potential conflict between self- versus group-relevance of stereotypical information, we also predicted that cognitive control regions, such as ACC and dorsolateral prefrontal cortex (DLPFC) (Gehring and Knight, 2000; Kim et al., 2010), could be implicated. If group membership is salient and a trait is descriptive of older adults in general but not of the self, older adults should experience conflict. Activation in control regions could allow an older adult to respond in a resilient manner. We hypothesized that ACC and DLPFC, implicated in cognitive control and conflict resolution, would show larger activations for resilient responses relative to threatened responses for older adults. We also expect ventral ACC and amygdala to exhibit greater activation for threat relative to resilient responses because threat responses should evoke a greater need for emotional processing. Younger adults’ neural activity should not differentiate resilient from threatened responses, as stereotypes should not elicit the need for conflict resolution in the case of a resilient response, or emotional processing in the case of a threat response.

Taken together, this study had three main goals. First, we investigated potential age differences in self-referential processing of stereotyped information, focusing on cortical midline regions associated with self-relevance. Second we assessed whether older adults exhibited a more social-comparison/contextual self-focus, reflected by greater activation than young adults in posterior regions, as opposed to an inward-directed self-focus when making decisions about stereotyped information. Third, we examined the neural basis of resiliency and stereotype threat for older compared to younger adults, predicting that brain activations implicated in conflict resolution and emotional processing, respectively, would underlie these two response types.

## Materials and Methods

### Participants

Seventeen young (ages 18–35) and 16 older adults (ages 66–83) participated in this study in exchange for compensation. Sample characteristics are presented in Table 1. One additional older participant was unable to complete the fMRI portion of the experiment due to discomfort in the scanner. Criteria for fMRI participation included right-handedness, English as a native language, good neurological, psychological, and physical health, and no CNS-active medication or other contraindications for MRI. The Brandeis University and Partners Healthcare Institutional Review Boards approved the study, and all participants provided written informed consent.

**Table 1:** **Means and standard deviations for demographics and performance measures**.

	Young	Elderly	*p*-Value
Age	23.41 (4.40)	76.25 (5.01)	0.001
*N*	17 (7 male)	16 (6 male)	
Years of education	15.53 (1.83)	16.63 (2.50)	0.16
Self-rated health	4.00 (0.79)	4.25 (0.78)	0.37
Digit comparison	80.41 (14.99)	55.81 (10.03)	0.001
Shipley vocabulary	34.47 (3.57)	36.63 (2.96)	0.07
MMSE	N/A	28.44 (1.31)	

Self-rated health reflects a rating on a 5-point scale, in comparison to others of one’s own age group. A rating of 4 denotes “better than average.”

### Neuropsychological measures

Each participant completed a health and demographic questionnaire, a digit comparison speed of processing task (Hedden et al., 2002) and a vocabulary task (Shipley, 1986). Older adults completed the Mini-Mental State Exam (MMSE; Folstein et al., 1975) in order to assess the orientation of the elderly participants. All elderly participants scored 27 or higher (out of 30) on the MMSE, as a means to include only cognitively intact older adults. Scores from these measures are presented in Table 1.

### Materials and procedure

Stimuli consisted of 216 trait adjectives. Seventy-two were stereotypical of older adults; half were positive (e.g., wise) and half were negative (e.g., frail). One hundred forty-four were control words and not stereotypical of either age group, with half positive (e.g., friendly) and half negative (e.g., irrational). Stereotypical words were taken from previously normed materials (Mueller et al., 1986; Bargh et al., 1996; Levy, 1996, 2003; Matheson et al., 2000; Boduroglu et al., 2006). Stereotype words were then assigned two unique control words from Anderson’s (1968) word norms and Affective Norms for English Words (ANEW; Bradley and Lang, 1999), matched on valence, word length, and word frequency based on Kucera–Francis and Throndike–Lorge measures of written frequency. Valence was determined for each stereotype word using Anderson’s word norms (Anderson, 1968). Words that were not present in Anderson’s word norms were assigned valence based on the valence of a root word or using the ANEW (Bradley and Lang, 1999). The distribution of trial types across the different conditions, broken down by endorsement, is presented in Table 2.

**Table 2:** **Means and standard deviations for the number of responses of each type at encoding**.

	Control		Overall	
	Yes	No	Missed responses	
Young	53.0 (7.8)	39.6 (8.1)	4.2 (5.2)	
Old	45.8 (6.7)	44.9 (7.7)	6.6 (8.1)	

	**Stereotype**			
	**Positive**		**Negative**	
	**Yes**	**No**	**Yes**	**No**

Young	19.7 (2.7)	3.8 (2.7)	7.3 (3.8)	16.4 (3.9)
Old	20.1 (3.8)	3.2 (3.5)	4.6 (2.8)	18.8 (2.9)

The experiment was presented using E-Prime software (Psychology Software Tools, Pittsburgh, PA, USA) and responses were recorded using a MRI-compatible button box. Before entering the scanner, participants were trained on the experimental tasks. The experimenter read instructions out loud while the participant read along, and then verbally confirmed understanding of the task. Participants completed a short practice session and were allowed to ask clarification questions. Once in the scanner, participants viewed 144 trait adjectives (96 control half positive, half negative and 48 stereotype words half positive, half negative) and judged whether each word was self-descriptive (e.g., “Are you compassionate?”). Stimuli appeared for 3 s with an additional second in which to make a response, followed by 2–20 s of fixation in a jittered design. For each trial, participants indicated a “yes” response using their index finger or a “no” response using their middle finger of their right hand. The 144 trait adjectives were split into three runs, each lasting 5 min. The entire scan session lasted approximately 45 min.

Approximately 10 min after the end of the encoding trials, participants were presented with surprise self-paced recall and recognition tasks outside of the scanner. These data will not be presented here, as they are not the focus of the current investigation. Before being debriefed and compensated for their time, participants completed a feedback questionnaire and an adjective rating sheet, in which they rated the extent to which adjectives described younger versus older adults. These ratings verified that both younger and older adults rated the stereotype words as more descriptive of older adults compared to the control words.

### fMRI acquisition

A Siemens Avanto 1.5 T scanner was used to acquire all structural and functional scans. An echo-planar imaging (EPI) sequence (TR = 2000 ms; TE = 40 ms) acquired 26 AC/PC oriented 5 mm thick slices (with a 1 mm skip between slices). Stimuli were projected onto a white screen behind the scanner, which the participant viewed through a mirror mounted to the headcoil. Participants who needed vision corrected wore MRI-compatible glasses. High-resolution structural images were acquired using a multiplanar rapidly acquired gradient echo sequence (MP-RAGE).

### fMRI analysis

Data were analyzed using Statistical Parametric Mapping (SPM8; Wellcome Trust Centre for Neuroimaging) implemented in MATLAB R2012a (The Mathworks Inc., Natick, MA, USA). The first five volumes of each session were discarded to allow for equilibration effects. The resulting EPI volumes were corrected for differences in slice time acquisition, using the middle slice of each volume as a reference, and spatially realigned to the first acquired volume to correct for movement. Each participant’s structural scan was coregistered to the mean EPI image produced from the realignment step and subsequently segmented and normalized to the Montreal Neurological Institute T1 average brain template. These normalization parameters were then applied to every EPI volume. The normalized EPIs were resliced into 3 mm × 3 mm × 3 mm resolution then spatially smoothed using an 8 mm full-width at half-maximum Gaussian kernel.

Analyses of the functional data from the study were carried out in two steps. In the first step, neural activity was modeled as a series of delta functions for each participant, coinciding with onsets of the various stimuli types convolved with a canonical hemodynamic response function. For each participant, 12 covariates were created, representing the 8 conditions of interest, 1 for “No Response” trials, and 3 representing each of the functional runs. Voxel-wise parameter estimates for all covariates were obtained by restricted maximum-likelihood (ReML) estimation, using a temporal high-pass filter (cutoff 128 s) to remove low-frequency drifts. Intrinsic autocorrelation within each session were corrected by applying a first-order autoregressive, AR(1), model. The data were scaled to a grand mean of 100 over all voxels and scans (Friston et al., 2007).

In the second analysis step, contrasts of the parameter estimates for each participant were submitted to a group analysis treating participant as a random effect. For each subject, we modeled four trial types: Stereotype Yes, Stereotype No, Control Yes, Control No. This lead to a 2 × 2 × 2 (word type: stereotype/control × decision: yes/no × age: young/old) mixed model ANOVA. A second ANOVA examined effects of resiliency and threat to the stereotyped words. To do this, we modeled positive stereotype words endorsed (i.e., “yes” responses at encoding) and negative stereotype words that were denied (i.e., “no” responses at encoding), together into a trial type called resilience. We grouped positive stereotype words that were denied and negative stereotype words that were endorsed into a trial type called threat. All words were processed in a self-referential manner. It is possible that all decisions made were objective, i.e., all “yes” responses were for words that were actually self-descriptive, and “no” responses were for words that were truly not self-descriptive, but in accordance with previous literature (Pinquart, 2002 – resiliency; Steele and Aronson, 1995 and other studies – threat) both resiliency and threat can have effects on performance that can cause participants to respond differently than they would in a non-experimental setting. We therefore presume that a threat versus resiliency response to a certain trial would be best characterized by the aforementioned grouping of trial types.

In all ANOVAs, eight group contrasts modeling the mean across conditions for each of the 33 participants were also added to each model to remove between-subject variance of no interest. Statistical parametric maps (SPMs) were created from the *T*-statistics for the various ANOVA effects of interest, using a single pooled error estimate for all contrasts, whose non-sphericity was estimated by ReML, as described in Friston et al. (2002). Results for each ANOVA are reported from two-tailed *t*-contrasts, threshold at *p* < 0.001, uncorrected with a minimum cluster size of 5.

## Results

Consistent with our focus on the effects of aging on self-referential processing of stereotyped information, we conducted three sets of contrasts.

### Age × stereotypicality

There was an interaction between age and word type with regions showing higher activation for stereotype words than for control words for older relative to younger adults ^2^. These effects emerged in posterior midline regions, including precuneus (BA 23, 7) and bilateral lingual gyrus (BA 18, 37). These regions were implicated in self-referential judgments about duties and obligations, a type of self-relevant agenda that remains highly motivating for older adults (Mitchell et al., 2009). All regions with significant activations can be seen in Table 3A. Although we also expected to see anterior midline frontal activations (such as mPFC) for this contrast, no activation emerged using the above threshold. Examining the reverse contrast to identify regions showing higher activation for control words than for stereotype words for older compared to younger adults yielded no significant activations. Neither did the main effect of word type (stereotype or control) yield any significant activation.

**Table 3:** **Age × stereotypicality**.

Contrast/region	Hemisphere	MNI coordinates			BA	*t*-Value	Cluster size
**(A) OLD > YOUNG (STEREOTYPE > CONTROL)**							
Middle temporal gyrus	Right	45	−70	16	39	4.46	71
Calcarine	Left	0	−79	10	17	4.36	122
Superior temporal gyrus	Right	63	−25	1	21	4.19	34
Cerebellum	Right	12	−46	−8	N/A	3.72	35
Lingual gyrus	Left	−18	−70	−2	18	3.63	13
Rolandic operculum	Right	54	−1	7	48	3.61	5
Inferior temporal gyrus	Left	−48	−58	−11	37	3.58	13
Lingual gyrus	Left	−24	−46	−8	37	3.58	11
Precuneus	Right	6	−52	46	N/A	3.52	6
Precuneus	Right	9	−58	22	23	3.5	5
Inferior frontal	Left	−27	38	−8	47	3.44	5
Precuneus	Right	12	−64	40	7	3.44	6
Fusiform	Right	24	−79	−11	18	3.42	7
**(B) OLD > YOUNG (CONTROL > STEREOTYPE)**							
No surviving voxels

*Regions are listed in order from highest to lowest t-value. Only one peak voxel is listed per cluster. BA, Brodmann’s area*.

### Age × stereotypicality × endorsement

**Figure 1:**
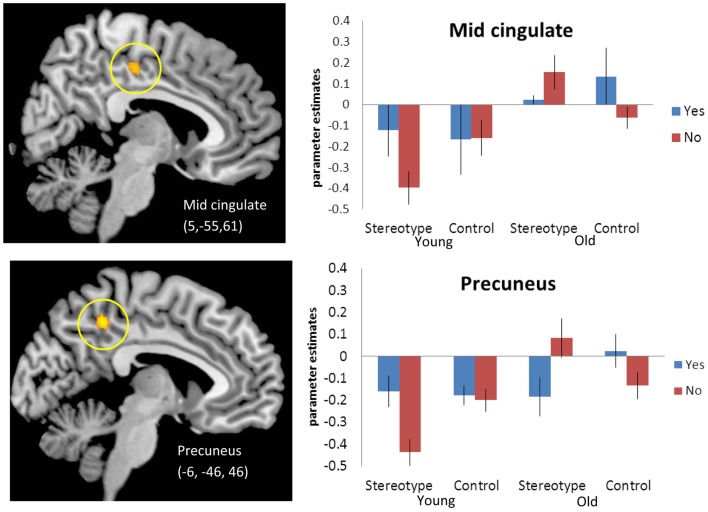
**[(SN-SY) > (CN-CY)], Older adults > Younger adults**. Threshold – *p* < 0.001, uncorrected, five contiguous voxels. Regions in which the difference in activation between stereotype words that were denied (SN) and stereotype words that were endorsed (SY) was greater than the difference between control denials (CN) and control endorsements (CY) for older adults compared to younger adults. Mid-cingulate activation (Top), Precuneus activation (Bottom).

We next tested for regions that in older adults, compared to younger adults, activated more for stereotype words that were non-endorsed (“no”) than for stereotyped words that were endorsed (“yes”) relative to control words [(SN-SY) > (CN-CY), Older > Younger Adults ^3^]. Thus, regions that for older adults responded more for rejected stereotype words than endorsed stereotype words, relative to endorsed versus rejected control words. Regions of activation surviving this contrast can be seen in Table 4A. Activations included posterior midline regions, including bilateral precuneus (BA 5) and right mid-cingulate (BA 23), as well as left amygdala. Figure 1 depicts the response for the right mid-cingulate, and left precuneus. The left precuneus showed differential activation across age, particularly for “no” stereotype words, with younger adults showing decreased activity and older adults showing increased recruitment. Similar effects were evident in the mid-cingulate and the amygdala (data not shown). We also tested for regions that emerged in the opposite contrast, with older adults showing higher activation for the “yes” stereotype words than the younger adults, but no significant activations were found (Table 4B).

**Table 4:** **Age × stereotypicality × endorsement**.

Contrast/region	Hemisphere	MNI coordinates			BA	*t*-Value	Cluster size
**(A) OLD > YOUNG [(SN-SY) > (CN-CY)]**							
Precuneus	Left	−6	−46	46	N/A	4.78	46
Supramarginal gyrus	Left	−60	−34	28	48	4.53	33
Mid-cingulate	Right	6	−22	46	23	4.2	25
Precuneus	Right	9	−55	61	5	4.05	17
Superior parietal	Left	−24	−73	46	19	3.99	18
Supramarginal gyrus	Right	60	−43	28	48	3.87	45
Insula	Left	−36	−10	−2	48	3.66	16
Putamen	Left	−24	11	10	48	3.6	15
Amygdala	Left	−21	2	−11		3.56	6
Insula	Left	−33	23	1	47	3.53	6
Precuneus	Left	−6	−43	61	5	3.44	7
Superior temporal gyrus	Left	−45	−43	22	41	3.39	5
Middle temporal gyrus	Left	−60	−22	−2	21	3.37	5
**(B) OLD > YOUNG [(SY-SN) > (CY-CN)]**							
No surviving voxels

*Regions are listed in order from highest to lowest t-value. Only one peak voxel is listed per cluster. BA, Brodmann’s area*.

### Age × threat/resiliency

**Figure 2:**
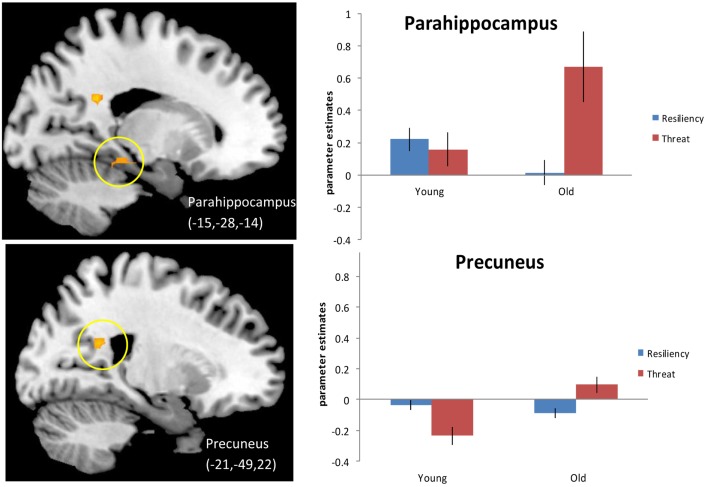
**Threat > Resiliency, Older adults > Younger adults**. Threshold – *p* < 0.001, uncorrected, five contiguous voxels. Regions in which the activation for threatening trials (defined as positive denials and negative endorsements) was greater than for resiliency trials (positive endorsements and negative denials) for older adults compared to younger adults. Parahippocampal gyrus for this contrast (Top), the slice displayed corresponds to *x* = −18 precuneus activation (Bottom).

We next examined activity during “threatening” trials (which we define as either “no” to stereotype positive words or “yes” to stereotype negative words) compared to resilience trials (defined as either “yes” to stereotype positive words or “no” to stereotype negative words) for older adults compared to younger adults (see Materials and Methods for explanation of trial groupings) ^4^. Principal regions emerging from the analysis again included posterior midline regions, such as left PCC (BA 23) and right precuneus (BA 17,7), as well as left hippocampus and left parahippocampal gyrus (BA 30) and are illustrated in Figure 2 and listed in Table 5A. The effect in the precuneus was driven by increased activation in older adults for threatening trials and decreased activation for resilience trials. The activation in PCC is centered in white matter so we cannot definitively say that it is related to recruitment of the PCC during the judgment task rather than an artifact. The effects in the hippocampus (data not shown) and the parahippocampal gyrus were driven by increased activation during threatening trials compared to resilience trials in older adults. Younger adults were generally insensitive to trial type, showing similar activation in these regions regardless of trial type, although they exhibited reduced activity in the precuneus in the threat condition. An examination of regions showing higher activation for resilience trials than for threatening trials (Table 5B) produced no significant effects.

**Table 5:** **Age × threat/resiliency**.

Contrast/region	Hemisphere	MNI coordinates			BA	*t*-Value	Cluster size
**(A) OLD > YOUNG (THREAT > RESILIENCY)**							
Posterior cingulate	Left	−15	−46	25	23	5.24	33
Middle temporal gyrus	Right	45	−67	22	39	4.64	43
Precuneus	Right	21	−49	22	17	4.48	25
Superior temporal gyrus	Right	63	−13	1	22	4.17	28
Parahippocampal gyrus	Left	−15	−28	−14	30	4.11	28
Fusiform	Right	30	−34	−14	37	4.06	7
Precuneus	Right	12	−67	55	7	3.81	10
Middle occipital gyrus	Left	−36	−61	1	37	3.71	6
Hippocampus	Left	−24	−37	4	N/A	3.68	5
**(B) OLD > YOUNG (RESILIENCY > THREAT)**							
No surviving voxels

*Regions are listed in order from highest to lowest t-value. Only one peak voxel is listed per cluster. BA, Brodmann’s area*.

## Discussion

This study used a self-referencing paradigm in order to investigate the neural regions involved at the intersection of thinking about oneself and age-related stereotypes. Given that older age represents one of the few stereotyped groups in which one transitions from an out-group member to an in-group member over the course of one’s life, this domain represents an opportunity in which to study how thinking about oneself is impacted by membership in a stereotyped group. In addition, the study explored the neural basis of stereotype threat and resiliency across age groups, suggesting that the processing of stereotyped information is impacted by the implications of endorsing it as self-relevant (e.g., reflecting a positive or negative self-view). Our first finding was that judgments of age-related stereotype words led to higher activations of posterior midline regions implicated in self-related processing, including precuneus and lingual gyrus, for older compared to younger adults. Second, older adults exhibited higher activity in precuneus, mid-cingulate, and amygdala for non-endorsed (non-self-relevant) stereotype words versus endorsed stereotyped words, compared to younger adults. Third, we showed that threat (i.e., denial of positive and endorsement of negative age-related stereotypes as self-relevant) relative to resilient responses (i.e., denial of negative and endorsement of positive age-related stereotypes as self-relevant) elicited increased precuneus, PCC, hippocampus, and parahippocampal gyrus activity. These findings converge in implicating changes to the posterior midline regions with age, suggesting that age groups may differ in thinking about the self in a highly contextualized manner during the processing of stereotyped information, particularly when information may be threatening to the self.

We predicted that midline cortical activity, indicative of self-referential processing, would be increased among older adults relative to young, in judging the self-descriptiveness of stereotyped versus control trait adjectives. We found age differences in posterior cortical midline regions (precuneus, lingual gyrus) that have been implicated in self-reflection, self-relevant memory, and other types of self-judgments (Johnson et al., 2006; Northoff et al., 2006; Gutchess et al., 2007; Mitchell et al., 2009). In particular, PCC activity increases as a function of self-relatedness (Moran et al., 2006) and posterior regions respond to thinking about duties and obligations (Johnson et al., 2006; Mitchell et al., 2009). Interestingly, stereotyping research with young adults also shows that the precuneus is more engaged for stereotype than control conditions (Quadflieg et al., 2009), though this has not been the focus of the literature thus far. We find that the effect in posterior regions emerges for older more than younger adults. While it was surprising to not identify effects in anterior midline regions (e.g., mPFC) given prior work, previous studies reporting frontal midline activation for self-referential processes (e.g., Kelley et al., 2002; Gutchess et al., 2007, among others) used experimental designs that included trials in which participants made non-self-referential judgments (e.g., judgments of other people or semantic judgments). This likely gave them more sensitivity to detect self-specific regions of activation. A possible explanation for why we did not see more activation of regions typically seen in self-referencing studies is that our experimental paradigm required participants to make decisions only in reference to the self, and so there was no other or semantic condition with which to compare. While the present study focused on midline cortical regions, it is worth noting that regions of superior temporal gyrus, located near the temporo-parietal junction, also exhibited age differences for stereotyped words, in comparison to control words (see Table 3, as well as Table 4). This region has previously been implicated in mentalizing and theory of mind (Van Overwalle and Baetens, 2009), suggesting that stereotyped words differently evoked processes involving in thinking about, and possibly empathizing with others, for older versus younger adults.

Our second hypothesis was that judgments of stereotype trait adjectives would necessitate a more outward social-comparison focus in self-referencing for older adults, due to the relevance of age-related stereotypes. We therefore expected that older adults would recruit posterior midline regions for judgments about age-related stereotype words more than control words, particularly when words were endorsed. The results of the aging × stereotypicality × endorsement contrast indicate that some posterior midline regions are sensitive to the self-relevance judgment of stereotyped information, such that there is a heightened response when older adults reject stereotyped information as non-self-relevant. Given the salience of age for stereotyped words, judgments about the self may evoke processing of the self in a social context, and this may be most salient when the judgment about the self differs from the expectation for the group (i.e., a “no” response to a stereotype). This explanation converges with some of our prior work in which we found that older adults engage precuneus more than young adults during the processing of pictures of social affiliation, whereas the groups similarly engaged the region for pictures of isolation (Beadle et al., 2012). Thus, the increased precuneus activity in older adults may reflect the tendency for age-related stereotypes to evoke more social processing in older than younger adults when the concept of the self versus the group is activated. It is also possible that the response reflects the threatening nature of the non-endorsed stereotyped information, as such words represent a poor outcome of aging that could limit one’s ability to perform duties and obligations. Such an interpretation would be consistent with the engagement of the amygdala and insula during this comparison, reflecting differential involvement of emotional processes across judgments.

Our third prediction was that threat responses would be subserved by activations in regions associated with emotional processing and emotional load, such as ventral anterior cingulate and amygdala, and that regions implicated in control processing and conflict resolution, including ACC and DLPFC, would underlie resilient responses for older adults (Gehring and Knight, 2000; Kim et al., 2010). Younger adults were expected to show no difference across response types. While we did not find any regions that were recruited significantly more for resilient responses over threat responses, we found that posterior midline and medial temporal regions (i.e., precuneus, parahippocampal gyrus, and hippocampus) showed increased activation for threat response trials compared to resilient responses for older adults relative to younger adults. This pattern is particularly interesting given that there were fewer threat trials compared to resilience trials and that old and young did not significantly differ in the numbers of trials per bin. However, the threat trials led to robust activation, particularly in the parahippocampal gyrus, for older adults. The engagement of parahippocampal gyrus during autobiographical memory tasks (Spreng et al., 2009; St. Jacques et al., 2011), taken together with the engagement of the hippocampus, could indicate older adults’ recall of specific episodic memories or scenes during threatened responses. As previously mentioned, precuneus has been implicated in self-referencing, particularly when thinking about the self in an outward-focused manner. This pattern could reflect that thinking about the self in a highly contextualized manner serves some protective function during threatening situations. For example, thinking about times in which one behaved in a manner consistent with a stereotype of old age could be considered situation-dependent, rather than as something typical of oneself. It is also possible that older adults are drawing on their richer store of autobiographical memories for times in which their behavior was stereotype-consistent. It is also interesting to consider whether the threat-related activity here reflects older adults’ over-activation of default regions during tasks. Older adults experience more difficulty suppressing default regions during externally driven tasks (Persson et al., 2007; Park et al., 2010), and one reason might be because the experimental conditions activate stereotype threat, and hence more activity in these cortical midline regions. Such an effect would have implications for a number of studies in the field of cognitive aging ^5^.

One of the largest limitations to our study was our inability to look at effects of valence due to the impoverished number of positive stereotype trials receiving a “no” response and negative stereotype trials receiving a “yes” response. While we combined across valences to create our measures of threat and resiliency, it would be helpful to separately examine the response to negative versus positive stimuli, particularly as negative stereotypes might be expected to drive the effects. Small bin sizes also prevented us from performing subsequent memory analyses to correlate brain activation during successful encoding, which would have allowed us to assess the effects of stereotypes on cognitive processes. Administering additional behavioral measures to substantiate the concepts of “threat” and “resiliency,” as well as self and peer-perception measures pre- and post-task (see Pinquart, 2002), could be combined with fMRI data to further explicate the function served by brain regions recruited during resilient and threatened responses, and individual differences as a function of one’s views of the self and aging.

In conclusion, we have shown that older adults process age-related stereotype words in a qualitatively different manner from younger adults, with different conditions eliciting more or less activity in regions for each age group. Older adults exhibit a more social-comparison/contextual self-focus when making decisions about stereotyped information, particularly in response to threat, as reflected by increased modulation of posterior midline regions. We have shown the possibility of dissociating resiliency from threat responses to stereotype information at the level of brain activation, suggesting that older adults may differently harness cognitive resources as a result of one’s personal views about the self and membership in a stereotyped group. This could indicate protective effects of seeing the self in a positive light, when compared to same age peers, which could impact cognitive function. Our data indicate that the neural regions engaged in response to stereotyped information can be influenced by the extent to which the information represents a threat or challenge to one’s self-image. These results illustrate the effects of aging on posterior, but not anterior, cortical midline regions during self-referential thought, and highlight the importance of understanding the effects of aging across the domains of self-reference and stereotyping.

## Conflict of Interest Statement

The authors declare that the research was conducted in the absence of any commercial or financial relationships that could be construed as a potential conflict of interest.

## Footnotes

^1^While research on stereotype threat does investigate the threat represented to the self, that literature focuses on performance decrements, rather than mentalizing about the self. Thus, this research will be reviewed elsewhere.

^2^Note that the contrast of (Old > Young) (Stereotype > Control) is equivalent to the contrast of (Young > Old) (Control > Stereotype).

^3^Note that the contrast of [(SN-SY) > (CN-CY), Old > Young] is equivalent to the contrast of [(SY-SN) > (CY-CN), Young > Old Adults].

^4^Note that the contrast of (Old > Young) (Threat > Resiliency) is equivalent to the contrast of (Young > Old) (Resiliency > Threat).

^5^We thank Reviewer 2 for this insightful point.
